# IGF-1, the Cross Road of the Nutritional, Inflammatory and Hormonal Pathways to Frailty

**DOI:** 10.3390/nu5104184

**Published:** 2013-10-21

**Authors:** Marcello Maggio, Francesca De Vita, Fulvio Lauretani, Valeria Buttò, Giuliana Bondi, Chiara Cattabiani, Antonio Nouvenne, Tiziana Meschi, Elisabetta Dall’Aglio, Gian Paolo Ceda

**Affiliations:** 1 Geriatric Rehabilitation Department, University Hospital of Parma, Via Gramsci, 14, Parma (PR) 43126, Italy; E-Mails: francy_devita@hotmail.it (F.V.); flauretani@ao.pr.it (F.L.); antonio.nouvenne@alice.it (A.N.); gpceda@unipr.it (G.P.C.); 2 Department of Clinical and Experimental Medicine, Section of Geriatrics, Food Sciences Unit and Endocrinology of Aging Unit, University of Parma, Via Gramsci, 14, Parma (PR) 43126, Italy; E-Mails: valeria.butto@studenti.unipr.it (V.B.); b.giuliana09@libero.it (G.B.); tiziana.meschi@unipr.it (T.M.); elisabetta.dallaglio@unipr.it (E.D.A.); 3 Azienda USL Piacenza, Via Taverna, 49, Piacenza (PC) 23121, Italy; E-Mail: chiarac2004@libero.it

**Keywords:** ageing, IGF-1, selenium, magnesium, zinc, micronutrients, frailty

## Abstract

The decline in functional capacity is a heterogeneous phenomenon in the elderly. An accelerated ageing determines a frail status. It results in an increased vulnerability to stressors for decreased physiological reserves. The early identification of a frail status is essential for preventing loss of functional capacity, and its clinical consequences. Frailty and mobility limitation result from an interplay of different pathways including multiple anabolic deficiency, inflammation, oxidative stress, and a poor nutritional status. However, the age-related decline in insulin-like growth factor 1 (IGF-1) bioactivity deserves special attention as it could represent the ideal crossroad of endocrine, inflammatory, and nutritional pathways to frailty. Several minerals, namely magnesium, selenium, and zinc, appear to be important determinants of IGF-1 bioactivity. This review aims to provide an overview of the potential usefulness of nutrients modulating IGF-1 as potential therapeutic targets in the prevention of mobility limitation occurring in frail older subjects.

## 1. Introduction

Aging is characterized by multiple changes in physiological pathways that concur to a decline in the functional capacity of older individuals. This is a heterogeneous phenomenon that predicts a large number of adverse outcomes including functional limitation, decreased cognitive function, reduced quality of life, and nursing home placement [1,2]. It is widely recognized that changes in physical performance and cognitive status are strongly predictors of mortality, independently of other traditional indicators of disease. Several aspects related to the impairment of functional capacity are included in the current definition of frailty proposed by Fried and colleagues [3]. Frailty represents a biological syndrome of increased vulnerability to stressors resulting from decreased physiological reserves. The main features of frailty are not well defined, but are likely to include an accelerated decline in physical function where sarcopenia, defined as the presence of both low muscle mass and low muscle function (strength or performance), is one of its most important clinical correlates [4,5]. Recent studies have demonstrated that frailty is a geriatric syndrome occurring in the early stages of mobility impairment and is not a synonymous or consequence of co-morbidity or disability.

In the elderly, frailty and mobility limitation, result from an interplay of multiple factors; the alteration of hormonal levels, pro-inflammatory state, oxidative stress, and nutritional status, seem to be the main important pathways of this complex phenomenon.

The multiple hormonal anabolic deficiency occurring with age, and characterized by a decline in dehydroepiandrosterone sulphate (DHEAS), testosterone (T), and insulin-like growth factor 1 (IGF-1), represents a peculiar mediator of mobility impairment in elderly population [6,7].

However, changes in GH/IGF-1 axis bioactivity deserve special attention since this axis is involved in the integration of endocrine, immune, and nutritional pathways. IGF-1 is an anabolic hormone that plays an active role in the maintenance of muscle mass and strength, in preventing apoptosis and in the protection from oxidative stress [8]. Both the secretion and the biological actions of IGF-1 are also modulated by the main pro-inflammatory cytokines [9]. Moreover, IGF-1 has been shown as a sensitive marker of nutritional status [10], especially in the elderly. In this context, IGF-1, due to its unique characteristics, may be assumed as an ideal cross-road of nutritional, hormonal, and inflammatory pathways to frailty. Interestingly, minerals, namely selenium, magnesium, and zinc have been shown to exert beneficial independent actions on muscle function and IGF-1 levels, playing a potential role in physical performance in the elderly. In our review we will particularly address the permissive role of each of these minerals on tissue production or liver secretion of IGF-1. Based on the current preliminary data, we will try to hypothesize a potential therapeutic implication of minerals in the modulation of IGF-1 bioactivity in frail older subjects at risk of mobility impairment.

The model of critical illness will be presented as an ideal context where the decline in IGF-1 levels occurring with age is worsened.

## 2. Insulin-Like Growth Factor

### 2.1. Insulin-Like Growth Factor Anabolic Hormone

IGF-1 is an anabolic hormone mainly synthesized in the liver and local expressed in peripheral tissues [11] under the control of pituitary growth hormone (GH). IGF-1 in turn seems to modulate GH secretion through a negative feedback mechanism [11]. GH/IGF-1 signaling is essential for normal growth in children and for the maintenance of anabolic processes in adults [11]. IGF-1 bioactivity and bioavailability are influenced by six binding proteins (IGFBPs), which also have independent biological actions [12]. The most abundant IGFBP in serum is IGFBP-3, which is a circulating IGF-1 reservoir with potential IGF-1-indipendent cell survival and proliferation effects [13]. During aging there is the gradual decline and alteration in GH secretion pattern and IGF-1 production. This phenomenon, called “somatopause” [14], contributes to a characteristic relative anabolic hormone deficiency in the elderly. The somatopause is associated to changes in body composition and metabolism similarly to those observed in adults with GH deficiency, namely a reduction of bone and muscle mass and strength, an increased fat mass, dyslipidemia, arterial hypertension, cardiovascular diseases, and cognitive decline [15]. The impaired activity of the GH/IGF-1 axis could be the basis of the multiple metabolic, biochemical, and functional changes that characterize the aging model [14]. As the relationship between the GH/IGF-1 axis and the musculoskeletal system is influenced by many variables, the results of clinical trials in humans are conflicting. Moreover, data regarding frail older subjects are very limited, and the hormonal cut-offs for IGF-1 associated with mobility limitation have to be established. Observational studies in frail elderly populations have shown a robust relationship between low circulating IGF-1 levels and sarcopenia, poor muscle strength, and physical performance. In healthy and frail older women (70–79 years old), low IGF-1 levels have been associated with poor knee extensor strength and self-reported difficulty on several mobility-related tasks but not with anthropometry or other strength measures [16]. In their study, Cappola *et al*. [16] showed a significant correlation between IGF-1 levels and muscle strength, only below IGF-1 threshold limit of 50 µg/L. Data from the Health, Aging and Body Composition Study showed a cross-sectional relationship between low circulating IGF-1 levels and poor thigh muscle area and density [17] in a cohort of elderly subjects of both sexes.

### 2.2. Insulin-Like Growth Factor and Inflammation

Interestingly, when both low IGF-1 levels and chronic inflammation are present there is a higher associated risk of progressive disability. Inflammatory cytokines increase with age, leading to a state of subclinical inflammation [18]. Upregulation of the inflammatory response plays a major role in the age-related decline of physical performance. Interleukin-6 (IL-6) is a key proinflammatory cytokine with negative effects on muscle function [19,20,21]. *In vitro* studies demonstrate that an over expression of IL-6 is one of the mechanisms implicated in the down-regulation of IGF-1 and IGFBP-3, suggesting that the negative effect of IL-6 on muscle function may be realized through IGF-1 [22]. Barbieri, Ferrucci *et al.* [23] evaluated the joint effect of IGF-1 and IL-6 on muscle function in a population-based sample of 526 participants, with a wide age range (20–102 years) from the InCHIANTI study, a prospective population-based study aimed at identifying the biological and clinical determinants of poor mobility and disability. They have documented the importance of inflammatory response in the decline of physical performance that occurs with aging. IL-6, IGF-1, and their interaction were significant predictors of handgrip and muscle power independent of potential confounders, such as age, sex, body mass index, soluble IL-6 receptor, and IL-6 promoter polymorphism. In analyses stratified by IL-6 tertiles, IGF-1 was an independent predictor of muscle function only in subjects in the lowest IL-6 tertiles, suggesting that the effect of IGF-1 on muscle function depends on IL-6 levels.

Recent data have confirmed that elevated levels of multiple catabolic biomarkers, including IL-6, are important predictors of the decline of muscle strength with aging [24]. In a cohort of 718 disabled older women, enrolled in the Women’s Health and Aging Study I [25], the group with low IGF-1 and high IL-6 levels had a greater risk for functional impairment compared with that having high IGF-1 and low IL-6 levels. In the Framingham Heart Study, composed of community-dwelling elderly subjects, an increased mortality rate was also observed in those participants with higher levels of TNF-α and IL-6, and low levels of IGF-1 [26]. Moreover, several lines of experimental evidence suggest that optimal circulating levels of IGF-1 are associated with a reduction of oxidative stress. Thus, IGF-1 may be implicated in various pathological conditions commonly associated with oxidative tissue damage [27]. It is known that free radicals exert adverse cellular effects interfering with oxidative phosphorylation [28].

### 2.3. Insulin-Like Growth Factor: A Sensitive Nutritional Marker in the Elderly

Nutrition seems to be a key regulator of circulating levels of IGF-1 [10]. A suboptimal nutrient intake negatively affects IGF-1 bioactivity and the anabolic milieu in the elderly. Nutrition regulates IGF-1 concentrations that may reflect changes in nitrogen balance induced by manipulation of nutrient intake. In humans, IGF-1 concentrations are reduced to the hypopituitary range after few days of fasting, and their normalization depends on the qualitative and quantitative composition of the refeeding diet [29]. In malnourished subjects undergoing repletion food, the increase in IGF-1 levels has been shown to be much stronger than changes of serum nutritional markers [29]. The decline of IGF-1 levels during dietary restriction seems to be independent of changes in pituitary GH secretion. Moreover, the role of the hepatic GH receptor does not seem to be related to the severity of nutritional insult. The marked reduction of the number of somatogenic receptors observed during fasting supports the hypothesis of IGF-1 receptor deficiency. In contrast, in selected forms of dietary restriction (protein restriction), the decline in IGF-1 is a consequence of a post-receptor defect in GH action at the liver level [29]. When an energy and/or protein deprivation occurs, there is a significant reduction in plasma levels of IGF-1 [30]. The dietary content of essential amino acids seems to be critical for optimal IGF-1 recovery after fasting. However, there is evidence of a threshold energy requirement under which the optimal protein intake fails to increase IGF-1 [31]. Holmes *et al.* [32], in their cross-sectional study population of more than one thousand female subjects, have shown significant lower IGFBP-3 levels in patients with higher fat intake, especially saturated fat. The hypothesis that IGF-1 is not only implicated in the regulation of cell growth, differentiation, and apoptosis, but it is also a potential and useful marker of malnutrition status, by defect or by excess, is supported by the U-shape relationship existing between IGF-1 serum concentrations and body mass index [33]. Lower IGF-1 levels are found during obesity, metabolic syndrome and diabetes [33]. Similarly, impaired IGF-1 levels have been documented in patients with HIV [34] and inflammatory bowel diseases [35]. In subjects undergoing protein-caloric restriction, it has been observed a significant reduction in circulating IGF-1 levels that has been correlated to the typical alteration in urea nitrogen urinary excretion occurring in malnutrition [36,37]. These data suggest that IGF-1 levels, reflecting acute directional changes in nitrogen balance and dietary energy, may represent a potential marker of nitrogen losses, useful for monitoring changes in protein metabolism In a study of malnourished patients with renal failure, IGF-1 was found to be a more sensitive indicator of malnutrition than classical nutritional markers such as albumin, pre-albumin, transferrin, and retinol-binding protein [38]. In children with growth failure due to chronic inflammatory intestinal diseases, IGF-1 has been used as marker of proper nutrition and reversibility of growth retardation [35]. An increased caloric intake produced an improvement in the levels of IGF-1 and human growth. The tight relationship between low levels of IGF-1 and malnutrition has been also described during celiac disease, where a rapid normalization of serum IGF-1 followed a gluten-free diet [39]. All these data support the potential usefulness of a positive modulation of IGF-1 levels especially in frail older individuals.

## 3. Minerals and IGF-1 in the Fire of Frailty

There is evidence suggesting that several minerals, namely magnesium, selenium, and zinc, along with optimal protein and caloric intake, could profoundly influence IGF-1 levels, IGF-1 bioactivity and the IGF-1 trophic actions on skeletal muscle. These minerals exert a wide range of pleiotropic functions on human cells and tissues playing a key role in the structure and function of the body.

**Figure 1:**
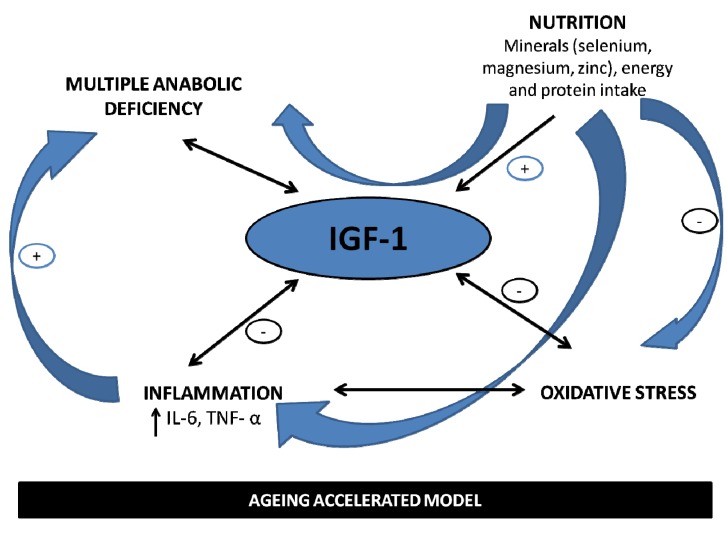
The central role of IGF-1 in the multiple pathways leading to increased frailty. IGF-1 is an ideal cross-road of hormonal anabolic milieu, nutritional and inflammatory statuses. IGF-1 levels are inversely related to inflammatory markers and oxidative stress and positively regulated by specific nutrients such as selenium, zinc, magnesium, along with energy and protein intake.

The interrelation between nutritional, hormonal and inflammatory pathways is especially evident in the ageing model as depicted in the Figure 1. Data suggest that a poor mineral status, frequently observed in older population, may exacerbate the age-related decline in IGF-1, influencing the multiple anabolic hormonal deficiency, one of main determinants of frailty.

We will try to describe in the following paragraphs that a mineral deficiency may trigger a low-grade chronic inflammation. This is especially true for magnesium and selenium. It is very well known that inflammatory cytokines act as negative regulatory signals that impair IGF-1 bioactivity and temper the action of other anabolic hormones and growth factors [40].

The interaction between immune and endocrine systems is necessary to maintain homeostasis. On the one hand GH and IGF-I regulate a variety of immune events [41] and, on the other hand, the inflammatory cytokines released from the innate immune system seem also to affect the neuroendocrine system [40]. The dysregulation of inflammatory response has been documented as a potential pathogenic factor in the development of several chronic inflammatory diseases, and of several phenomena of accelerating ageing, such as sarcopenia and poor mobility.

Moreover, a selenium deficiency could also promote oxidative stress and cellular damage by decreasing selenoproteins concentration, such as glutathione peroxidase, that in turn have been shown to affect IGF-1 bioactivity. Similarly the link between zinc and IGF-1 could be at least in part attributed to the known antioxidant activity of zinc. Selenium and zinc by protecting tissues, including the endocrine cells, from oxidative stress, could positively modulate IGF-1 cell-releasing by preventing cells degeneration and limiting the production of oxygen reactive species. Thus, these actions may exert an important role in the age-related inflammatory conditions.

The potential maintenance of GH/IGF-1 axis by minerals is also of importance because anabolic hormones do not operate independent of each other and might have synergistic effects. IGF-1 can be also considered the cross-road of many stimuli other than GH and including other anabolic hormones such as DHEAS and T [42]. All together, the anabolic hormones influence the anabolic nutritional state, the satellite cell activation and when combined with exercise and other mechanical stimuli exert a strong influence on muscle hypertrophy. The alteration of the GH/IGF-1 axis could, therefore, negatively contribute to the alteration of other hormonal pathways, worsening the overall anabolic state.

However, although the potential underlying mechanisms of the relationship between these micronutrients and IGF-1 have been proposed in preliminary cross-sectional analyses, this hypothesis has not been tested in randomized clinical trials (RCT).

### 3.1. Magnesium and IGF-1

Magnesium is an intracellular cation with a key role in the structure and function of human body [43]. It is involved in many metabolic pathways, in addition of being a cofactor in fundamental biological processes including protein and acid nucleic synthesis and neuromuscular excitability. Magnesium may directly bind and alter structure of enzymes (e.g., RNA and DNA polymerases), and exerts pleiomorphic activity as part of the activated Mg-ATP complex. It is also essential for the activity of all adenosine triphosphate and phosphate transfer-associated enzymes [43].

Epidemiological, experimental, and clinical studies support the presence of low magnesium intake and reduced serum magnesium levels in numerous clinical and preclinical conditions including defective membrane function, type-2 diabetes, metabolic syndrome, elevated C-reactive protein (CRP), hypertension, atherosclerotic vascular disease, increased oxidative stress, and immune dysfunction [44,45].

Interestingly, magnesium has been shown to exert, as IGF-1 does, beneficial independent actions on muscle function and could play a role on physical performance in the elderly. This hypothesis is consistent with clinical and epidemiologic data supporting the importance of the magnesium ion as a determinant of muscle performance in young subjects. Structural damages to muscle cells are associated to magnesium depletion and an optimal magnesium status seems to be necessary for maintaining muscle performance and exercise tolerance in young individuals, with evidence of significant increasing in muscle strength after magnesium supplementation [46,47]. However, the role of magnesium in maintaining muscle integrity and function has not been fully investigated in older adults. This is surprising given the higher prevalence of poor magnesium status in this specific population [48].

Dominguez *et al*. [49] found, in a representative cohort of older subjects, a significant independent and strong positive relationship between circulating magnesium levels and measures of muscle performance, including grip strength (β = 2.0 ± 0.5, *p* = 0.0002), lower-leg muscle power (β = 8.8 ± 2.7, *p* = 0.001), knee extension torque (β = 31.2 ± 7.9, *p* < 0.0001), and ankle extension strength (β = 3.8 ± 0.5, *p* < 0.0001).

There is also evidence that some of beneficial effects of magnesium on muscle function, especially in older population, could be exerted through the stimulation of IGF-1.

Maggio and colleagues [50] have investigated for the first time the relationship between magnesium and IGF-1 using a cohort of 399 older men ≥65 years of the InCHIANTI study. They found that magnesium levels were strongly and independently associated with total IGF-1 levels (β ± SE, 15.9 ± 4.8; *p* = 0.001).

In fasting normotensive subjects IGF-1 was able to increase intracellular magnesium levels in a dose- and time-dependent fashion also reversing the blunted response to insulin of hypertensive cells [51]. IGF-1 potentiates insulin-induced stimulation of magnesium at doses that, themselves, do not raise magnesium, supporting both the hypothesis of a role for IGF-1 in cellular magnesium metabolism and the importance of magnesium as a determinant of insulin action. This intriguing relationship might primarily depend of a poor magnesium status that, by increasing systemic oxidative stress and inflammation, may contribute to down-regulate IGF-1 secretion. Interestingly, magnesium, but also IGF-1, are strongly related with antioxidant capacity. IGF-1 levels rapidly fall after exposure to oxidative stress [28]. Moreover, magnesium deficiency has been associated with increased oxygen-derived free radicals, oxygen peroxide production, decreased antioxidant enzyme expression, and activity [52,53] and higher levels of interleukin 1-β, tumor necrosis factors-α, and IL-6 [54], suggesting again a potential mechanistic link between IGF-1 and inflammation.

Since magnesium status seems to be strictly related to muscle ATP and IGF-1 levels, it becomes important to check whether the contemporary presence of both magnesium deficiency and low IGF-1 levels can be related to age-related phenomena. It is possible that poor magnesium status contributes to the onset of late-life sarcopenia. Magnesium deficiency might exacerbate the age-related decline in IGF-1 levels, playing an additive negative effect in impairing physical performance. This is of particular importance in the older population, which is more vulnerable to develop frailty.

In the elderly, the reduced nutrient intake along with impaired magnesium absorption and/or increased urinary loss, as well as polypharmacotherapy, could explain why a poor magnesium status is frequently observed. Although magnesium requirement does not change with age [55], data from the National Health and Nutrition Examination Survey (NHANES) III have reported an age-related decrease in daily magnesium intake, that frequently does not reach dietary reference intake values (420 and 320 mg/day for men and women respectively) [56]. The ageing model is also characterized by a major risk of magnesium deficit due to the age-dependent fall of intracellular magnesium levels and the increasing in magnesium retention [57]. This phenomenon suggests a subclinical magnesium deficit in the elderly, which is difficult to be detected by total serum magnesium [58]. With age, there is a decline in magnesium absorption efficiency from the intestine and in tubular resorption at the renal level. This condition could be influenced by alterations of vitamin D metabolism or latent primary renal disorder, which is frequently observed in older persons [57]. It is also known that medications such as loop diuretics [59], digitals [59], and proton pump inhibitors [60,61] could interfere with magnesium absorption and excretion leading to a secondary magnesium deficiency.

These preliminary data require further investigations in order to confirm if magnesium could be assumed as predictor of anabolic hormones, such as IGF-1, and represents a potential therapeutic option in order to prevent mobility limitation in older persons.

### 3.2. Selenium and IGF-1

Selenium is a micronutrient with a wide range of multiple functions on cells and tissues by integration into 25 selenoproteins that have selenocysteine residues at their active core [62]. Selenoproteins are involved in the glutathione peroxidases family, a group of antioxidant enzymes [63]. Selenium is also an essential cofactor of many metabolic pathways. After being transformed into its bioactive metabolites, selenium acts on the nuclear transcription factor NF-κB [64], signal transduction, cell cycle checkpoint and apoptosis [65]. Selenium is implicated in the iodothyronine deiodinases function, enzymes on which depends the production of active thyroid hormone (triiodothyronine or T3) in the peripheral tissue [66,67]. Selenium status, measured by plasma or serum selenium, varies by country and corresponds to dietary intakes. Dietary selenium intakes ranges from 7 µg per day to 49,990 µg per day, with mean values of 40 µg per day in Europe and 1135 µg per day in USA, where 50% of the population takes dietary supplements [68]. Recommended selenium intake is 60 µg per day for men and 53 µg per day for women [68]. Food sources of selenium are meats and fish (typical selenium content 25–150 mg/kg), cereals and grains (typical selenium content 10–75 mg/kg), milk and dairy products (typical selenium content 10–25 mg/kg), and vegetables and fruits (typical selenium content 0–20 mg/kg) [69].

Rayman and colleagues in a recent review [70], analyzed a wide number of studies investigating the relationship between selenium status and human health. Low selenium status has been associated with increased risk of mortality [71,72,73] and cognitive impairment [67]. On the other side, optimal selenium status seems to exert a protective effect on viral infection [74], autoimmune thyroid disease [75] and cancer [76,77].

There is increasing evidence that selenium could influence skeletal muscle function even if its role in maintaining functional muscle efficiency is still unclear.

Selenium may contribute to skeletal muscle function through the maintenance of an optimal concentration of glutathione peroxidase and other selenoproteins, particularly the selenoprotein N, located in endoplasmic reticulum that seems to regulate calcium mobilization required for early muscle development [63].

Many studies have detected that selenium deficiency contributes to the pathogenesis of myopathies [78]. An important and surprising observation came from Moosman and Behl [79], who noticed that muscular symptoms associated with selenium deficiency are very similar to those often encountered during statin use. The authors suggested that molecules of the family of statins may interfere with the isopentenil tRNASec, inhibiting its maturation in functional tRNASec, and leading to a defect in the selenoproteins synthesis and low muscle function.

Beck *et al*. [80] have shown a significant positive association between selenium levels and handgrip values in a population of elderly women with moderate to severe levels of disability.

Lauretani *et al.* [81], analysing data form 891 participants (77.1% of all population enrolled) of the InCHIANTI study, found a linear association between plasma selenium levels and muscle strength, namely hip flexion and hip extension for lower extremity muscle strength, shoulder adduction and handgrip for upper-extremity muscle strength. After adjustment for age, sex, BMI, total energy intake, chronic diseases, and IL-6 levels, participants in the lowest quartile of plasma selenium concentration had a greater risk of poor knee, hip, and grip strength decline than those in the highest quartile. More than 30% of the study population presented selenium concentration <0.88 mmol/L (<70 µg/L), a level below which selenoproteins synthesis could be significantly reduced. It is possible that the reduced selenoproteins activity can increase stress and oxidative damage to DNA, proteins and cellular lipids. In the Uppsala Longitudinal Study of Adult Men [82], high serum levels of selenium were predictive of low concentrations of urinary F2 isoprostane, a marker of lipid peroxidation and oxidative stress. The accumulation of damage at the mitochondrial level and nuclear DNA is one of the main etiological hypotheses by which loss of myocytes and a consequent impaired skeletal muscle function is realized. Selenium, being an important cofactor in cellular protection, could be particularly involved in muscle protection, especially in the elderly where the cellular oxidative damage is more prevalent.

Recent findings support the hypothesis of a possible role of selenium in IGF-1 bioactivity. A recent cross-sectional study that involved 44 young women showed a significant association between IGF-1 serum levels and selenium from dietary sources (*r* = 0.41, *p* < 0.01) [83].

Maggio *et al.* [84], in a recent study have explored this hypothetical link in 951 participants (90% of enrolled) of the InCHIANTI Study. Factors statistically correlated with IGF-1 levels were identified and introduced in the multivariate analysis. This study found a strong correlation between selenium and IGF-1 levels in older adults that was independent of age, sex, body mass index, total energy and alcohol intakes, smoking, alanine aminotransferase, thyroid stimulating hormone, free thyroxine, and free triiodothyronine, CRP, IL-6 and the major chronic diseases (congestive heart failure, chronic obstructive pulmonary disease, and cancer). This finding raises the hypothesis that selenium is capable of modulating IGF-1 bioactivity also at skeletal muscle level.

The hypothesis of a link between selenium and IGF-1 is supported by the observation of a fine balance between selenium and GH in ensuring normal growth. Animal studies suggest that selenium deficiency is independently associated to growth retardation, compromising triiodothyronine availability and GH/IGF-1 axis [85].

Long-term sodium selenite treatment in rats reduced serum GH levels, IGF-1 and IGF binding protein-1, -2, and -3 and determined a growth delay [86]. The underlying mechanism of this phenomenon has not been fully elucidated in the study, but was probably caused by the accumulation of selenium in secreting vesicles that inhibited GH secretion. Moreover, a selenium deficiency causes an increased T4 (67%) and a reduction of T3 (23%). By using second-generation rat pups with selenium deficiency, maintaining adequate vitamin E and methionine levels, administration of 0.1 or 0.2 mg/g selenium normalized thyroid hormones serum levels, selenium liver content, and glutathione peroxidase activity [86]. The exclusive T3 administration did not restore the growth, suggesting that factors other than serum T3 might be involved in growth-related disorders.

These findings are particularly interesting since IGF-1 and selenium play a significant role in the pathophysiology of some age-related phenomena such as sarcopenia. A common target for selenium and IGF-1 is the skeletal muscle tissue. As previously described, both selenium and IGF-1 are involved in muscle protein synthesis and their deficiencies may lead to sarcopenia and disability during aging. Moreover, both low selenium and IGF-1 levels are associated with anemia, another potential determinant of sarcopenia [87,88,89].

The positive association between selenium levels and IGF-1 primarily depends on the positive action exerted by selenium levels on IGF-1. Selenoenzymes such as glutathione peroxidase and glutathione reductase protecting tissues from oxidative stress [82] could positively affect IGF-1 cell-releasing. Selenoenzymes seem to prevent cell degeneration and oxygen reactive species production. An alternative hypothesis to explain the relationship between selenium and IGF-1 considers inflammation as the common negative regulator of these two factors. In fact, selenium deficiency worsens during increased NF-κB activity [64] and by this mechanism can determine the up-regulation of IL-6. In turn, IL-6 could negatively influence IGF-1 levels. In Women’s Health and Aging Study I, subjects with low levels of selenium had higher levels of IL-6 [90]. As previously described, a similar inverse association was also found between IGF-1 and IL-6 [23]. We cannot also ignore the role played by dietary intake. Primary dietary sources of selenium are proteins [69], which are also important positive modulators of serum IGF-1 concentrations [31].

Future longitudinal and intervention studies will clarify the mechanisms of the intriguing association between selenium and IGF-1 and the effects of dietary and supplementary selenium sources on IGF-1. These data will create the rationale for a replacement treatment combining selenium and IGF-1 in the prevention and treatment of sarcopenia and frailty.

### 3.3. Zinc and IGF-1

Zinc is an essential micronutrient with a structural and functional role in a large number of macromolecules and enzymatic reactions. More than 200 enzymes implicated in major metabolic processes require zinc as a functional component. Zinc is involved in growth, protein and DNA synthesis, neuro-sensory functions, cell-mediated immunity, thyroid function, and bone metabolism [91]. Suboptimal zinc status is associated with impaired wound healing, dermatitis, inflammation, and depressed immune response and might be responsible for the high incidence of age-related infections and degenerative diseases [92]. Interestingly, zinc seems also to be involved in nutritional regulation of IGF-1 bioactivity. In cultured bone cells, some studies suggest that zinc potentiates the action of IGF-1 [93] and increases endogenous IGF-1 synthesis [94]. In animal models, severe zinc deficiency has been shown to decrease hepatic IGF-1 gene expression and to impair intracellular GH signaling pathway [95,96,97]. By these mechanisms, zinc may affect circulating concentrations of insulin, IGF-1 and GH. Roth and Kirchgessner [98] showed that force-feeding a zinc-depleted diet to rats for 14 days results in a 28% decrease in serum IGF-1 compared with rats fed a zinc-adequate diet, although there was no difference in caloric food intake. Similarly, Droup *et al*. demonstrated that low IGF-1 levels were related to decreased zinc concentration [99]. However, maintaining serum IGF-1 levels by exogenous administration or by inducing food intake, or both, in zinc-deficient rats, was not sufficient to correct the growth inhibition induced by zinc deficiency [100]. Thus, zinc could be involved in some aspects of growth regulation at the cellular level independently of those observed on circulating IGF-1 and GH. Changes in the GH-IGF axis alone might not explain the growth inhibition observed in zinc deficiency. In humans, poor zinc status has been associated with low circulating IGF-1 concentrations even in presence of adequate caloric intake [101]. In zinc deficient short children, zinc supplementation was effective in inducing growth. In these subjects, the growth velocity was associated with increased plasma IGF-1 concentrations [102,103,104]. These data suggest that growth-stimulating effect of zinc might be mediated through changes in circulating IGF-1. However, when optimal serum zinc levels are present in children with idiopathic short stature, zinc supplementation increased basal IGF-1, IGFBP-3, alkaline phosphatase, and osteocalcin levels but did not significantly change height or weight standard deviations during a 6–12 month follow-up period [105]. Thus, zinc supplementation may be beneficial in selected categories of subjects with low baseline zinc serum levels. A moderate zinc deficiency is also often observed in elderly subjects [106]. During ageing, nutritional deficits coupled with decreased absorptive efficiency contribute to zinc depletion. More evidence suggests that an increased rate of bone resorption with age is an important negative determinant of zinc excretion. In postmenopausal osteoporotic women has been reported a parallel decline of bone zinc and calcium, with a marked hyperzincuria [107,108,109,110]. A zinc deficit could exacerbate the age-related decline in IGF-1 serum levels. However, despite suboptimal zinc intake is widely reported in the elderly, the relationship between zinc status and IGF-1 has not been adequately investigated. Devine *et al*. [111] examined the relation between caloric intake and IGF-1 and IGFBPs concentrations in 119 postmenopausal women. IGF-1 concentrations were significantly correlated with mean protein and zinc intake at baseline (*r* = 0.313, *p* = 0.001; *r* = 0.298, *p* = 0.001, respectively) and after two years (*r* = 0.256, *p* = 0.008; *r* = 0.331, *p* = 0.001, respectively), even after adjustment for age, weight, and other nutrients. In sixty-one hospitalized frail elderly aged 66.7 to 105.8, with a mini-nutritional assessment score between 17 and 24, Rodondi and colleagues [112] determined the effects of dietary zinc intake (30 mg/day) on IGF-1 and bone turnover responses to four-week essential amino acids-whey protein supplementation After one week, zinc supplementation increased the IGF-1 response to essential amino acids-whey protein (+48.2 ± 14.3 and +22.4 ± 4.7%, *p* < 0.05), and significantly decreased markers of bone resorption.

The link between zinc and IGF-1 can be at least in part attributed to antioxidant activity of zinc. *In vitro*, animal and human studies show that zinc decreases the oxidative damage of membrane fractions probably through a protection of sulphhydryl groups against oxidation and the inhibition of the production of reactive oxygen by transition metals [113,114,115]. Although zinc may act as a biological antioxidant, high levels of zinc could also be a pro-oxidant agent by eliciting a decline in erythrocyte Cu–Zinc superoxide dismutase (SOD) [116]. Moreover, zinc is provided by protein foods, especially animal products [117], known to increase IGF-1 serum levels. In population of healthy well-nourished middle-aged and elderly men, Larsson and coworkers [118] tested the association between total energy, alcohol, vitamins, protein, nutrient-dense core foods (including red meat, fish and seafood, poultry, and milk), and serum IGF-1 concentrations. Interestingly, these authors observed a statistically significant positive association between protein intake, zinc, and serum IGF-1 concentrations independent of age.

However, zinc assessment is difficult because a sensitive, specific biomarker for this mineral has not been identified yet [119]. Plasma (or serum) zinc concentration, being responsive to both zinc supplementation and zinc depletion, seems to be the most widely reported biomarker for assessing zinc status. However, data at this regard are not very strong. Hair and urine zinc concentrations are also considered useful biomarkers of zinc supplementation [119].

Dietary zinc recommendations vary widely across Europe due to the heterogeneity of approaches used by expert panels [120]. The determination of zinc requirements is traditional based on a factorial approach that estimates the zinc intake required to meet physiological requirements for growth, metabolism and tissue repair while replacing obligatory losses. An alternative approach is based on the examination of the dose–response relationship between intake and biomarkers of zinc status and also between intake and health outcomes. The relationship between zinc and IGF-1 in ageing population warrants further investigation in order to confirm whether zinc could be identified as a modulator of IGF-1 bioactivity in frail older persons at risk of mobility limitation.

## 4. Perspectives

**Figure 2:**
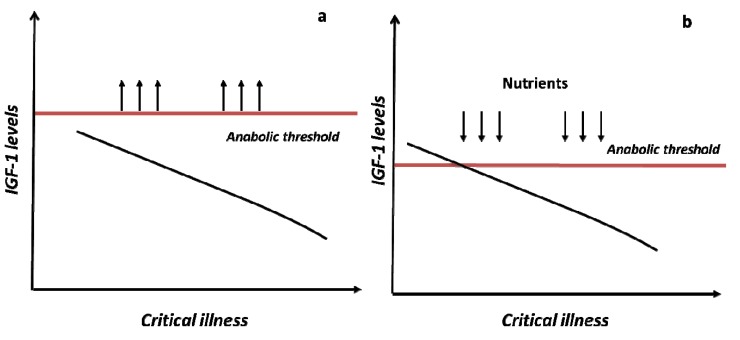
Potential therapeutic implication of mineral supplementation during critical illness.

Based on the data presented here it can be hypothesized a potential therapeutic implication of mineral supplementation or replacement in well-defined groups of older persons, and particularly in those with a decreased physiological reserve and increased vulnerability to stressors. These subjects are more prone to develop mobility limitation, and often show a suboptimal nutritional status and lower baseline IGF-1 levels. The perfect context where micronutrients might be useful is the model of critical illness. Stress, acute illness, surgery or trauma produce major changes in the metabolic milieu of the body such as altered substrate utilization and synthesis, hypermetabolism, and catabolism [121,122,123]. The robust response of the anterior pituitary gland, cytokines, and the sympathetic nervous system at the outset of critical illness dramatically changes over time. Older frail individuals have a reduced ability and require more energetic costs to recover from acute stressors with prolonged acute critical illness and chronic critical illness [124]. Severe muscle atrophy, weakness, and disability are direct consequences of this reduced homeostatic reserve [125]. During an acute critical illness, cortisol, as well as other counter-regulatory hormones and cytokines, support the inflammatory response and vital organ function [126] shunting substrate away to promote anabolism. Impaired IGF-1 levels along with the rise in GH, augments the lipolytic and insulin-antagonizing effects of GH, while postponing the anabolic effect of IGF-1 [126]. In prolonged acute and chronic critical illnesses the loss of pulse GH amplitude profoundly affects IGF-1 levels, which tend to drop further, worsening the catabolic milieu of the critically ill older patient [126]. The resulted higher anabolic threshold to stress is particularly important in very frail elderly subjects, characterized by lower IGF-1 levels and inadequate nutritional status even before the acute events. Moreover, acute critical illness is also followed by a prolonged bed rest that emphasizes the catabolic process and favors the onset of disability [127]. The use of certain micronutrients as modulators of IGF-1 levels and anabolic threshold might be an intriguing future therapeutic option. This concept it is graphically depicted in Figure 2.

In both panel, a and b, the lines represent the decline in IGF-1 levels which is realized during a critical illness. In panel a, the higher anabolic thresholds resulting from prolonged acute and chronic critical illness is described. Panel b underlines the potential therapeutic implication of mineral supplementation in lowering such anabolic threshold.

## 5. Conclusions

The age-related gradual decline in IGF-1 levels is part of the multiple anabolic hormone deficiency. This phenomenon could be implicated in the development of sarcopenia, mobility limitation, and frailty syndrome. IGF-1 is a sensitive nutritional marker rather than just an anabolic hormone and is negatively influenced by poor mineral and global nutritional statuses and subclinical low-grade inflammation. These conditions may influence the physical performance in older individuals by exacerbating the negative anabolic milieu. IGF-1, by representing a crossroad between hormonal, inflammatory, and nutritional pathways of frailty, might contribute to an earlier interception of those subjects more sensitive to tailored interventions. The increasing evidence that magnesium, selenium, and zinc along with optimal protein and energy requirement, are implicated in sustaining IGF-1 levels is of importance especially in elderly population.

These preliminary findings suggest that both, minerals and IGF-1, could be considered specific therapeutic targets to improve muscle strength and physical performance in the elderly. Longitudinal studies and RCT are needed to test the precise contribution of multiple mineral deficiencies to frailty, and to establish the specific cut-offs of micronutrients and the optimal dosages required to increase the anabolic status and physical function in older individuals. The findings of these upcoming studies will create the rationale for an effective combined approach in the prevention and treatment of frailty and other conditions of accelerated aging.
